# A Rare Case of Hypohidrotic Ectodermal Dysplasia in a Seven-Year-Old Child

**DOI:** 10.7759/cureus.24300

**Published:** 2022-04-20

**Authors:** Syed Asfand Yar Shah, Wajih Ul Hassan, Waseem Sajjad, Usama Bin Shabbir, Ali Raza

**Affiliations:** 1 Internal Medicine, Nishtar Medical University, Multan, PAK

**Keywords:** heat intolerance, hypodontia, anhidrosis, midface hypoplasia, ectodermal dysplasia

## Abstract

Ectodermal dysplasias (EDs) encompass a large group of inherited disorders that affects two or more ectodermally derived structures. Hair, sweat glands, teeth, and nails are the most common ectodermal derivates affected. Other ectodermal structures that may be affected are ears, eyes, lips, and mucous membranes of the mouth or nose. During embryonic development, the ectoderm forms the outermost layer of the primary germ layers that give rise to the several structures that are commonly affected in ED. Therefore, ED manifests differently among patients, depending on the abnormality's combination and severity. Out of 150 distinctive syndromes, the most common syndromes within this group are hypohidrotic (defective sweat glands) and hidrotic (normal sweat glands). In addition, different types of inheritance patterns are found in ED; X-linked inheritance is by far the most common mode of inheritance. We present here the clinical case of hypohidrotic (anhidrotic) ED in a seven-year-old boy.

## Introduction

Ectodermal dysplasia (ED) is not a single disorder but a complex group of various genetic disorders that primarily have an effect on the skin and its appendages. More than 180 different types of ED have been identified [1]. Depending on the particular type (syndrome), an ED can also affect the skin, the eyes or ears, the lining of the airways, the development of fingers and toes, the nerves, and other parts of the body [1].

Two major groups can be distinguished based on the presence or absence of sweat gland defects: (1) hypohidrotic or anhidrotic (Christ-Siemens-Touraine syndrome), in which sweat glands are absent or significantly reduced in number, and which appear to have an X-linked inheritance pattern; (2) hydrotic (Clouston syndrome), in which sweat glands are normal and which has an autosomal dominant inheritance pattern.

Hereditary Hypohidrotic ED (HHED) is an X-linked recessive Mendelian character that is usually seen in males and inherited through female carriers. Males are affected severely, while females show only minor defects [2]. More than 192 diseases have been discovered in this condition, with an estimated incidence rate of seven occurrences per 1,000 infants [3]. Chondro ED (Ellis Van Creveld syndrome), Cranio ED (Sensenbrenner syndrome), Incontinentia Pigmenti, Ectrodactyly-ED-Clefting Syndrome, and Rapp-Hodgkin ED are some of the more frequent syndromes. Others may have autosomal dominant or recessive traits, but hypohidrotic types have an X-linked recessive trait.

Even though beard development in affected males may be normal, scalp and body hair are usually dry, thin, scanty, and hypopigmented. In addition, hair growth is slow and infrequent, and it may be overly brittle, unruly, or crooked. Nails are commonly thick, irregular, discolored, and brittle. The skin may be lightly pigmented. Rashes and infections are common, and the skin on the palms and soles can be thick (hyperkeratosis). One of the distinctive features of ED, on which a diagnosis is commonly established or confirmed, is abnormal tooth development resulting in missing teeth (hypodontia) or small pointed coned-shaped teeth. In many cases, a complete set of permanent or deciduous teeth is missing (anodontia). In addition, because of inactive proteins in the sweat glands, their sweat glands may operate improperly or may not have evolved at all. As a result, the body cannot regulate temperature effectively without normal sweat production. Therefore, overheating is a typical issue, particularly in hot weather.

People with ED frequently exhibit particular cranial-facial characteristics that might be distinguished: frontal bossing is common, longer or more pronounced chins are common, and broader noses are also common. The objective of this study was to report a case of HHED in seven-year-old boy.

## Case presentation

A seven-year-old boy presented in the outpatient department in Nishtar Medical University Hospital, Multan, with a chief complaint of heat intolerance. He also had a history of dry skin, delayed teeth eruption, and loss of hair growth.

Parents revealed that the child has an excessive heat intolerance issue and finds it difficult to deal with the hot weather in summer. To cope with this condition, he takes a bath several times a day. The patient had consanguineous parents and two siblings. His eight-month-old brother has a similar problem and requires multiple baths a day; however, his sister is normal, having no such symptoms.

**Figure 1:**
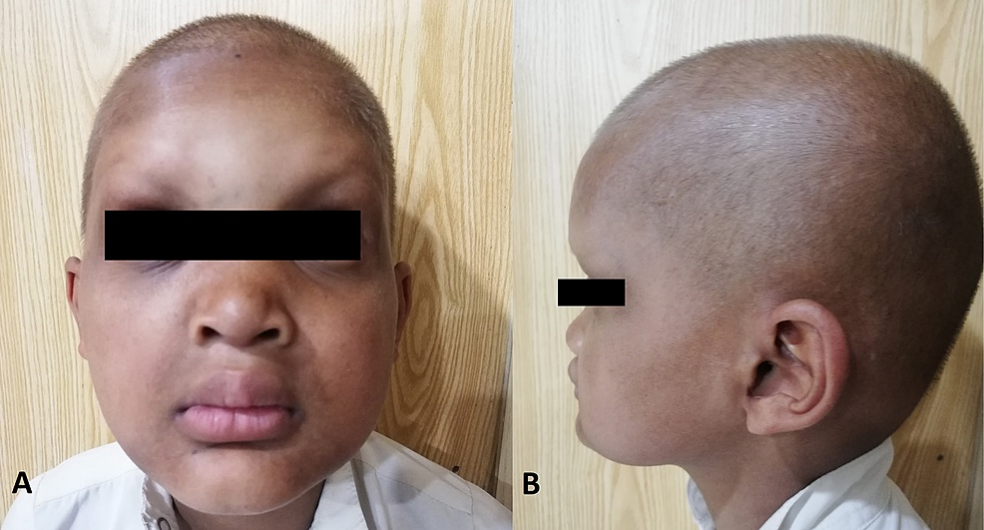
Clinical presentation of Ectodermal dysplasia. (A) Frontal view. (B) Lateral view Photograph showing dry skin with decreased skin color (pigment), large prominent forehead, low nasal bridge, thin sparse hair, minimal eyebrow, and thick lips.

Upon examination, the patient was vitally stable. He had frontal bossing, depressed nasal bridge, thick lips, sparse golden/brown hair on the scalp, scanty eyebrows, and hyperpigmented spots on the skin. The patient also has periorbital and perioral wrinkling (Figures 1A, 1B). Nails were normal with no significant hand or foot deformity.

**Figure 2:**
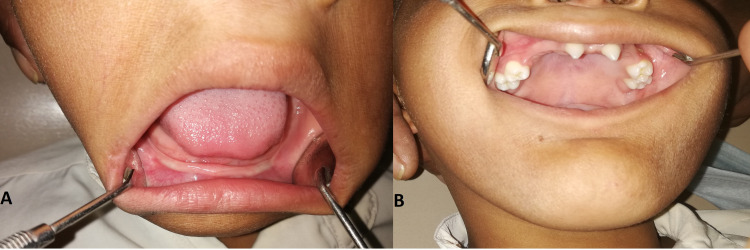
Intraoral examination. (A) Mandibula arch. (B) Maxillary arch Photograph showing extra orally dry skin with perioral wrinkling and intraorally conical-shaped maxillary central incisors with mandibular anodontia.

Intraoral examination shows a total absence of mandibular teeth and maxillary hypodontia. Two central deciduous incisors and bilateral deciduous molars were seen in the maxillary arch (Figures 2A, 2B).

**Figure 3:**
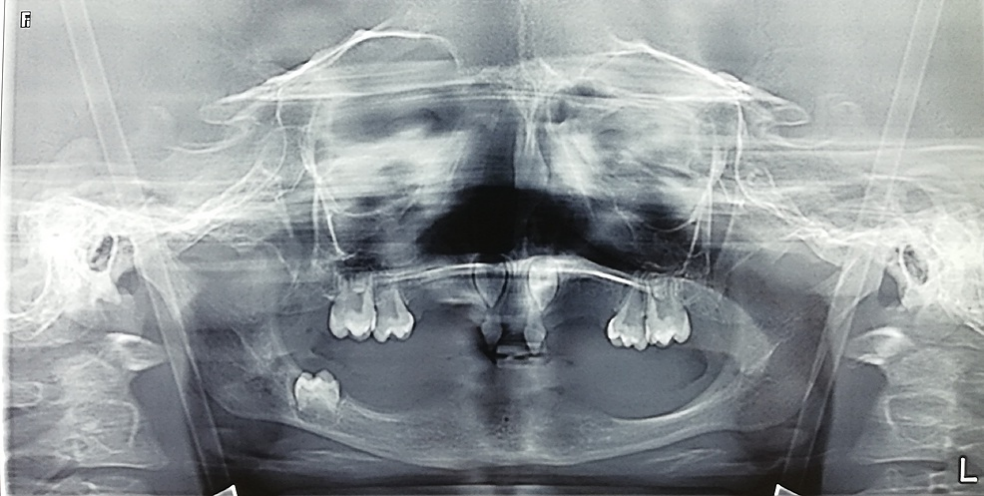
Orthopantomogram shows the absence of teeth in the mandible.

Orthopantomogram shows the absence of teeth in the mandible except for eruption of a right-sided first deciduous molar, presence of only upper primary central incisors, and bilateral maxillary deciduous molars (Figure 3).

He was given an emollient with topical steroid for his eczematous rash and sunscreen for use over the sun-exposed area before going under the sun. In addition, parents were educated about the disease and were advised to observe precautions regarding vigorous physical activities and exposure of the child to the hot environment. Light cotton clothing and cotton bed linen were recommended. He was also advised to keep good oral hygiene and avoid sun exposure. Furthermore, he was referred to an orthodontic specialist for expert dental management.

## Discussion

ED represents a large group of hereditary disorders characterized by congenital defects of one or more ectodermal structures, including skin appendages. The prevalence of ED is believed to be around seven per 10,000 births, and all mendelian modes of inheritance have been documented [4]. The disorder might occur during the first trimester of pregnancy. If it is severe, it appears before the sixth week of embryonic life, and consequently, the dentition will be affected. After the eighth week, other ectodermal structures may be affected [5].

Hair abnormalities are the most common manifestation (>90% of patients), followed by dental problems (80%), nail defects (75%), and sweat gland malformations (42%) [6]. Enamel hypoplasia, microdontia, and partial to complete tooth loss are examples of dental anomalies. In addition, hyperkeratotic palms and soles are prevalent, as are pseudo-rhagades around the eyes. A large forehead with prominent supraorbital ridges, saddle nose, low set ears, a depressed midface, a lack of alveolar bone growth in the lower third of the face, and protuberant lips are all common facial traits [7].

The gene responsible for X-linked HED is localized at Xq12-q13.1 and affects a transmembrane protein expressed by keratinocytes, hair follicles, and sweat glands, possibly having a key role in epithelial-mesenchymal signaling [8]. On the other hand, hidrotic ED is inherited only by the autosomal dominant pattern by changes in the GJB6 gene, encoding connexion‑30, and located in chromosome 13 (locus 13q11‑q12) [9]. Molecular studies have found that the abovementioned genes are responsible for the formation of several substrates required for the activation of the tumor necrosis factor α‑related signaling pathway, the WNT‑signaling pathway, and the nuclear factor‑κB pathway, involved in ectoderm‑mesoderm interactions, differentiation of ectodermal appendages, and organogenesis during the initiation of embryonic development [10].

The key to determining intrafamilial genetic transmission is an assessment of relatives of patients with hypohidrotic ED and pointing out the carriers of partial forms of the disorder in their families [11]. Suppose, there was no other occurrence of ED among the relatives. In that case, it implies that the propositus was most likely caused by a new mutation or gene translocation, as stated in some previous research [5].

Mutations in EDA/EDAR/EDAEADD can be detected to establish an early prenatal diagnosis in participants with a family history of hypohidrotic ED using DNA-based linkage analysis and genetic assays [12]. Sonography and fetal skin biopsy are both good diagnostic tests for the second trimester of pregnancy [13].

Morbidity and mortality in patients with hypohidrotic ED mainly depend on the function and number of sweat and mucous glands, and it also puts the patients with hypohidrotic ED at the risk of upper respiratory tract infection [14]. No definite pharmacological treatment is available, and the management of affected patients depends on which structures are involved [14]. Children with decreased sweating may have a mortality rate of up to 30% in infancy or early childhood because of intermittent hyperpyrexia [15].

Because there are so many clinical and psychological factors to consider in patients with hypohidrotic ED, a multidisciplinary approach is essential. Patients with hypohidrotic ED and hyperpyrexia may present to the treating physician. Cold sponging, frequent showers, cooling vest, using cooled emollients, drinking cold water, and other cooling techniques are necessary for such patients and should be started instantly.

Parents should be counseled and instructed to keep their children away from strenuous physical activities and exposure to extreme heat. Atopic dermatitis, xerostomia, and dryness of the eyes and nose are other common ED signs that should be addressed symptomatically [12,16]. All hypohidrotic ED patients should be sent to a dentist. Patients with hypohidrotic ED are vulnerable to depression, low self-confidence, and insecurity because of their odd physical features and lack social acceptance [12,16].

The administration of recombinant EDAA1 intravenously to newborn dogs with X-linked hypohidrotic ED has been shown to improve the growth of their skin tissues, teeth, and mucous glands [17]. In addition, intraamniotic administration of recombinant EDAA1 to pregnant mice enhanced the phenotype of the X-linked hypohidrotic ED in newborn mice [18]. Recombinant EDAA1 is currently in Phase two clinical studies, where it is being given to newborn males with HHED in the hopes of alleviating some of their symptoms.

## Conclusions

EDs are a diverse group of hereditary illnesses with overlapping features that make classification challenging. Clinical manifestations pose major social problems in affected persons. Early diagnosis and rehabilitation through interdisciplinary joint efforts are usually required. The patients often need to be treated by a team of doctors including pediatricians, ENT specialists, and expert dentists. Moreover, the help of psychologists is generally required to help ED patients retain their self-esteem.
